# Plantation of Teeth

**Published:** 1896-03

**Authors:** Louis Jack

**Affiliations:** Philadelphia


### International Dental Journal.

Vol. XVII. March, 1896. No. 3.

### Original Communications.[1]

### PLANTATION OF TEETH.[2]

BY LOUIS JACK, D.D.S., PHILADELPHIA.

The planting of teeth is divisible into three classes: *Transplantation*, where teeth removed from one subject are with proper precautions placed in an alveolus of another immediately after the extraction of a diseased or fractured tooth. *Replantation*, where any given tooth of a person for various reasons may have been removed, and after certain manipulation of the tooth and treatment of the socket is replaced. *Implantation*, where an artificial alveolus is made by trephining the process at a vacant place, in which opening a suitable natural tooth is introduced.

*Transplantation* has been credited to Dr. John Hunter, but undoubtedly had been done before him. Directly after his demonstration of the feasibility of the procedure the operation was frequently performed in England, France, and to some extent in this country. The required conditions at that time were that the tooth to be transplanted should in size and form be as near as attainable the same as the one to be substituted, and necessitated one person, for a consideration, to yield the organ for the benefit of another.

[1] The editor and publishers are not responsible for the views of authors of papers published in this department, nor for any claim to novelty, or otherwise, that may be made by them. No papers will be received for this department that have appeared in any other journal published in the country.

[2] Read before the Academy of Stomatology, December 17, 1895.

The removal of the tooth and the substitution were made immediately, it being considered essential that the operation be done under conditions favoring direct union.

This operation soon fell into disuse for reasons which are now clear. Dangerous inflammation frequently occurred by the transmission of disease from one subject to another, and more frequently, probably by introduction of pathogenic germs, serious disorders, sometimes followed by death, ensued.

Apparently the first described attempts in this country of the transplantation of dried teeth which had been long removed appeared in a paper, by Dr. S. P. Cutter, read before the Odontological Society of New York, January 18, 1877. An abstract of cases is that two waste upper bicuspids were inserted side by side. After six months, on account of pain in the maxilla, the second bicuspid was removed, requiring considerable force to displace it. The first was permitted to remain.

CASE II.—A first bicuspid was removed; the roots broke off, were then drilled out, and the septum of bone between them drilled away. An old tooth was fixed in the socket which after ten months was firm.

Here, in passing, occurs not only the earliest attempt to use dried teeth, but also, by drilling the socket to change the form, the first suggestion of implantation appears.[1]

It should be noted here that John Hunter stated that many dentists prefer dead teeth, and, further, that he had seen dead teeth become perfectly firm after insertion, and do service for many years.

*Replantation* is not traceable to any authority for its beginning. It probably had its rise in the natural tendency to replace teeth dislocated by accident or removed by mistake. Our literature shows that for this purpose it was generally successful without the care which would now be exercised to insure a safe result. Within recent periods it has been performed as a means of effecting the cure of chronic alveolar abscess and other disorders of the root. The results have generally been reported as curative. This practice has been confined to the more careful and judicious practitioners, which is borne out by an examination of the literature of replantation.

Successful treatment in this manner and for this latter purpose became coincident with the scientific practice of aseptic surgery. Without careful consideration of the application of the principles

---

[1] For the report and discussion, see Dental Cosmos, vol. xix., p. 258 *et seq.*

governing asepsis, replantation in disordered condition of the teeth and peridental membrane would be as liable to failure and as dangerous as transplantation in the previous century.

The *rationale* of the treatment here would appear to be, if we exclude the influence of shortening the root and occluding the root-canal, that the traumatism induces for a time an acute or subacute condition for the previous chronic one.

The relation of failures in replantation and transplantation previous to 1885 should be ignored. A tooth extracted and handled necessarily becomes an infected thing, and without disinfection its presence in the tissues of the alveolus involves a continuous struggle between the vital forces of the part and the development of bacteria. But through all these influences occasionally replanted and transplanted teeth remained for many years. I knew of a case of a man who had an inflamed lateral incisor removed. The pulp had been devitalized. Deploring the loss, he replaced it on retiring for the night. On arising it had escaped. Not to be outdone, he tore from his handkerchief a narrow part of the selvedge, and with this tied the tooth to one of the adjacent ones. It remained for fourteen years.

*Implantation* was undoubtedly originated by Dr. Younger, whose claim has not been publicly disputed. The date of his first operation was June 15, 1885. The general procedures of this operation as practised by him are so familiar that it is needless here to repeat them.

The result of the fixation of an implanted tooth when it has been properly adjusted and held in position has become indisputable. The evidence presented by men of reliability and sincerity of purpose has too much accumulated to fail to be convincing as to the feasibility of implantation.

There are certain requirements already established which govern success. The artificial opening must have the depth to give correct occlusion, and be but little larger than the root of the tooth to be inserted. The opening in the gum should be smaller than the cross section area at the cervix of the tooth. The mouth should be disinfected. The operator's hands and all instruments must be put in an aseptic condition. The tooth should be rendered impersonal and aseptic by being heated in a disinfectant fluid. The tooth should not have shreds of peridental membrane upon it, but should not be scraped clean of the adherent portion of the membrane. During intervals of the operation the socket should be filled with a tent of the antiseptic fluid in use. When the tooth is inserted it is better that some force be employed to push it into place. It should then be securely fixed in position by some means which will give immobility.

With the exception of the formation of the opening in the gum and the trephining of the process, implantation has no essential differentiation from the requirements governing transplantation and replantation.

With this outline I will proceed to describe the methods I have pursued in the cases under my care.

The most important qualification connected with these operations is the means to secure thoroughness of asepsis. The usual reliance has been upon bichloride of mercury. I have avoided its use on account of its irritating influence and its depression of the vital force of the tissues, and have relied upon the aqueous solution of hydronaphtol and the aqueous solution of acetanilide.

The former is soluble in the proportions of 1 to 300. The latter is freely soluble in the ratio of 1 to 200. To effect the solution of hydronaphtol it is first dissolved in alcohol, when it becomes entirely miscible in water. In these proportions neither is irritating to the tissues, and when heated, thoroughly effect the disinfection of the tooth, the hands, and instruments. The importance of heating disinfectant solutions to increase their power has been well established. (For example, with bichloride of mercury 1 to 20,000 of urine at 104° F. prevents fermentation. Anthrax spores lose their infecting power in weak solutions of the ordinary antiseptics at 170° F.). In each case I place the tooth in either of the above disinfectant solutions and raise the temperature to the boiling-point. The instruments likewise are heated in the same manner. These and the tooth are kept in the fluid in the intervals which occur during the surgical procedures.

Generally I have subjected the selected tooth to this process for a short time after opening the pulp-chamber to disinfect the root-canals.

The importance of securing immobility of the planted tooth cannot be overestimated, since the structural changes which produce the union of the tooth cannot be expected without a state of rest. It must be as essential here as in the case of fractures, since the process of ankylosis must be analogous to the process of the union of bones. Therefore, I emphasize the necessity for absolute fixation. I take an impression in plaster, and upon the cast make a study of the case. The tooth is fitted in the plaster to correct relations with adjoining and the occluding teeth. It is then secured in place, when a metal cast is taken, and upon this is fitted a gold splint of whatever form and with whatever attachments may give stability. This is secured by clasps or by wire ligatures to the other teeth at the cervix. Additional security and cleanliness is effected by setting it in place with phosphate of zinc.

For the front teeth, which are the most difficult to fix, in some cases I open into the pulp-chamber on the lingual surface and insert a wire parallel with this face. The splint is formed to pass between the pin and the tooth. This, if the splint is secured to the adjacent teeth, establishes the fixation in each direction.

These mechanical preparations may appear to some to be unnecessarily tedious. I have to state upon this point that in a question where success depends upon careful observances that these precautions are essential. To protect the surface of the root from injury during these procedures it is covered with a strip of mucilage paper wrapped upon it, or a layer of floss silk. To prevent drying out, the cast is covered with damp paper or kept in a box among damp paper. The final operation upon the mouth is simple, and is speedily done. It is, as above stated, preceded by every aseptic precaution.

In respect of replantation and transplantation, I invariably have broken up the remains in the alveolus of the peridental membrane. My impressions have been that this places these cases in the same relations which occur in implantation. The grounds for this procedure are that the experience of those who have replanted and transplanted teeth were of very frequent failure by early resorption, while the implantations when carefully performed have given favorable results.

I have *implanted* four cases; of these, three became fixed with apparent calcific support.

No. 1. Left upper central, male; operation April 20, 1889. In a few months it became firm and remained so for six years and five months, when it suddenly became loose and fell away. At the time of the operation the patient was in good health, but for a year or more before the resorption he had failed in vigor.

No. 2. Right upper lateral, male; operation January 12, 1895. The tooth became attached quickly, and is now resonant.

No. 3. Right upper central, same patient as No. 2.; operation same date. On account of the occlusion of the lower tooth upon the tongue of the splint, secure attachment failed. It was removed December 7, 1895, when another tooth was inserted. It is notable that the alveolus had contracted as the root became diminished by resorption, and required deepening and enlargement to receive the new tooth. The location of these teeth had been edentulous for thirty years.

No. 4. Right upper first bicuspid, male; operation April 23, 1895. Progressed favorably; splint removed in four months. Tooth now very resonant.

My transplantations have been five.

No. 1. Left upper lateral, female; operation October 22, 1892. Tooth dead; deep pocket on palatal aspect; fistula on gum and labial aspect; apex of root much denuded of membrane and quite loose. Deepened the alveolus and firmly splinted the case; splint removed in five months; calcification was not established until four months afterwards. The tooth remains perfectly firm, and is very resonant.

No. 2. Right lower lateral; operation October 10, 1894. Profound pyorrhœa, with much resorption of margins of alveolus; patient not of good health; tooth became fastened by connective tissue, but it failed to become firm and was removed in six months. Here I was unable to attach a splint, and relied upon wire ligatures, which were not sufficient to prevent motion.

No. 3. Left upper lateral, male; operation January 12, 1895. The root here was deeply split, the parts much separated, thus enlarging the alveolus. The gum quickly embraced the root. In a few weeks the tooth became firm; it is now very resonant. Here the face became swollen, with perceptible heat, which quickly responded to treatment.

No. 4. Left upper cuspid, female; operation March 12, 1895. Here the tooth had been nearly disconnected by pericementitis; excessive flow of pus; outer plate of alveolus very much destroyed. The alveolus was fully sprayed with the solution of acetanilide; a tent also of lint saturated with an alcoholic solution of the same was applied for a few minutes. The next day swelling of the tissue about the tooth occurred. The face over the canine fossa was suffused and swollen; refrigerated face and sprayed gum with acetanilide one-half per cent. In two days these appearances subsided; no trace of pus could be elicited by pressure over the tooth. November 9, after eight months, the tooth being resonant, the splint was removed. This case is remarkable from the extent of resorption of the alveolus, and from the free suppuration consequent upon the depth of the pockets.

No. 5. Right upper second bicuspid, male. The tooth very loose from pericementitis; process much resorbed, but no discharge of pus at the time; tooth removed November 2, 1894; operation twenty days later. The fixation by splint was not secure from disturbance by the occlusion of the lower tooth. There is now no indication of calcification.

Of replantations I have had three cases.

No. 1. Right upper first bicuspid, female; operation November 3, 1894. Displacement outward by pericementitis, with resorption of outer wall of alveolus. Reformed the socket to secure alignment. In three hours replaced the tooth. Removed splint in six months. The tooth is now firm and very resonant.

No. 2. Right upper lateral, female ; operation January 19, 1895. Tooth disturbed outward and posteriorly, distorting the lip, caused by pericementitis. Deepened the alveolus and reformed it to secure alignment. Management same as in previous case. The gum became slightly inflamed, which passed away in six days. The splint was removed at end of June. Tooth firm and resonant.

No. 3. Right upper central, female ; a case of pyorrhœa with enlargement of the alveolus ; operation March 23, 1885. Here, because the remaining adjacent teeth were not firm, a state of immobility could not be secured. The ultimate attachment is doubtful.

The apparent promising results in teeth affected by the consequences of pericementitis is an important conclusion to be drawn from several of the above cases.

I have alluded to the means taken to keep the root clean and protect the surface from injury. If a protected root apparently free of portions of the peridental membrane be immersed in warm water or a disinfecting solution the surface slightly swells, becomes velvety to the touch, and is viscid. It is a rational conclusion that the plasma effused into the space surrounding the planted tooth must necessarily come into more intimate connection with the tooth in this condition than would be the case if the root were polished. Moreover, a tooth in the condition stated when removed during the operation for adjustment exhibits a considerable degree of resistance to displacement, which I have attributed largely to this condition.

Since the appearance of Dr. Oscar Amoedo's paper upon "Implantation of Decalcified Teeth," read before the International Medical Congress in 1894 (see *Dental Cosmos*, vol. xxxvi., p. 680), I have in each case partially decalcified the cementum, being careful not to carry the process so far as Dr. Amoedo recommended. The partial decalcification cleanses the surface, removes any slight traces of calculus and softens somewhat the surface, and is in line with the hypothetic reasons given above for the retention of the viscid coating of the pericementum. The decalcification is not done until after the previously described mechanical procedures, and only shortly before the insertion of the tooth and immediately after the final disinfection.

The question pertinently arises whether the remains of the cementum covering the root, along with the morphological change induced by the decalcification of the surface of the cementum, may not act as a matrix to assist the organization of the plastic elements effused. While it is not in agreement with histology that dead animal substance can become revivified, it is not an irrational hypothesis to account for organization of plasma taking place under the influence of the morphological arrangement of tissue which afterwards may become resorbed. In these cases, too, the teeth assume a freshness of color scarcely distinguishable from living teeth and much different from the tint they have after their disinfection and complete preparation. This phenomenon, which cannot be vital in its nature, indicates that the influence of the fluids of the part has a marked corrective effect upon the shade of the tooth.

The condition of the surrounding tissues after these operations presents a surprisingly small degree of disturbance. When inflammation appears it does not run high, is little disposed to become diffused, and quickly yields to mild refrigeration. The general view is that the absence of any inflammatory symptoms is salutary. This may arise from the dread involved in the word, based as it probably is on the disturbing effects of that condition common to the period previous to knowledge of the methods to secure asepsis. But when reparative action is desired a certain and limited degree of excitement of the neighboring tissues is necessary to induce the supply of the plastic element required, and to set in motion the cell-development which conducts the reparative effort which closes the breach of continuity. This would appear to coincide with what takes place in simple fractures, as, when the neighboring tissues are somewhat lacerated by the separation and displacement of the fractured ends of the bone, the amount of callus is more abundant than in pipe-stem fractures, when in some instances the bones fail to be sufficiently surrounded at the seat of the fracture, and are feebly supported during the completion of calcification. May not the same principle obtain here? If the effusion of plastic matter is slight, and confined to the space between the root and the wall of the alveolus, it is reasonable to infer that the calcific connection will be comparatively thin, whereas, if the inflammatory process were deeper and more acute than is here usual, the plastic matter would fill the contiguous alveolar spaces to greater depth, and therefore we should expect a larger zone of calcific deposit about the root.

It should be held in view in this connection that the cancellated portion of the alveolar process contains an extremely small amount of osseous tissue. In view of this fact it is important to the secure fixation of planted teeth, and of the permanence of their attachment, that the area of calcification should be extended beyond close proximity to the root of the planted tooth. Close observation, in connection with control of the secondary disturbances instead of alleviation of the inflammatory condition, is needed to establish this conclusion. If such a conclusion be reached it would appear correct to induce a greater disturbance of the tissues than is ordinarily produced by the operation.

We have in this connection the supporting fact that osteitis of the alveolar process not attended by formation of pus is followed by calcification of the cancellated portion, and that the ossification is persistent.

In my cases, where the inflammatory indications have been most apparent and longest continued the attachment has been earlier established and the ultimate resonance more marked than when no signs of secondary disturbance have been noticed.

The character of the union and the histological process by which this is brought about is the most important scientific consideration which is presented in connection with the plantation of teeth. Is it an encystment where the root is only tolerated until some local disturbance or constitutional depression occurs to bring about a retrograde process to expel the intruded tooth? or is it ankylosis where the osseous reproduction fuses the tooth to the neighboring lattice-like formation surrounding it?

The observations bearing upon these questions have not been conclusive. They have been made upon displaced teeth which have either failed to become united to their surroundings, from either local or other causes, or they have been upon teeth which, after calcification had been secured, have undergone resorption, and therefore have not told the character of the connection. We lack the observation of such teeth at their best results to determine the process of the repair of the breach between the root and the environment.

As all healing of wounds is by one method, that of organization, it is only necessary to bring to bear the observed facts which have been determined by the insertion in the tissues of pieces of dried lung, of sponge or of pith. There quickly appears congestion about the irritant, then exudation of fluid and solid elements; the former penetrates the pores quickly, the latter slowly. Next follows the development and proliferation of connective-tissue cells and the formation of granulated tissue with development of blood-vessels.

The connective-tissue cells undergo differentiation in accordance with the tissue which is to be formed or reproduced in reparation, and are variously named as fibroblasts, osteoblasts, odontoblasts, etc.

In the process of reparation under consideration another phase of it enters into view. When dried pieces of lung-tissue are inserted for experimental observation, as previously alluded to, the leucocytes during the process of organization remove all traces of these; but if the irritant is of such a nature as to be intractable to these, another element appears,—the giant cell, the function of which is eliminatory.

If the leucocytes have the ability to remove the viscid film of the peridonteum and to take up the small amount of exposed decalcified tissue, there would appear no difficulty in the organization of the osteoblasts and the embracement of the root by their prolongations. If, on the other hand, the remains of these structures will not be accepted by the former cells, the giant cells will remove them. When this action has taken place after plantation of teeth the process does not stop here, but the cementum is attacked. The views of Amoedo and others are that this is the necessary action, and that after a time this ceases and calcifications take place in the bays so produced, and thus by the subsidence of resorption and the deposit of a layer similar to cementum the tooth becomes fixed.

This to me is a problematical result, since in each such case I have observed that the surface of the root had been placed in a more irrritating condition by the acute margins and sharp speculation, so that reparation would appear questionable. I have therefore aimed to render the tooth as slightly irritating as possible, by careful preparation, by partial decalcification, by the use of mild and stimulating aseptics combined with the most absolute rest, and the continuance of this state until resonance appears.

It is unfortunate that in too many instances planted teeth, which have become fixed and useful organs, have after several years, from causes not entirely clear, yielded to resorption and have become useless. The termination of these cases seems to come on with suddenness and without warning of the resorptive process. The cause of this result we have to look for in a depressed trophic state of the environment, in which condition it is not unreasonable to expect tissue of repair to be the first to yield. In any defective condition of the nutrition of a part this result is in consonance with well-fixed principles.

We have also to consider that the alveolar process is a provisional structure, and that its tendency is, on the occurrence of irritation or of impaired nutrition or by static conditions, to yield to resorption. The fact that it is provisional would impress upon it the tendency to this structural change.

Notwithstanding the prognosis of planted teeth is at present precarious, the promise of useful operations is so large when selection is made of subjects in sound health and without cachectic taints that where plantation is required for the good of the patient, it should be performed. The probabilities are that greater experience will lead to improved methods and more certain results.

