# An Important Correction

**Published:** 1896-05

**Authors:** Louis Jack


### AN IMPORTANT CORRECTION.

To the Editor:

In the paper read upon "Plantation of the Teeth," before the Academy of Stomatology of Philadelphia, December 17, 1895, I credited Dr. Cutler with having made, in 1877, the first publication of the alteration of the alveolus to adapt it to the size and form of the tooth to be transplanted. This appears to be an error, since I have found later that in an article by Dr. Wm. N. Morrison, before the American Dental Association in 1876, and published in its transactions, he described the obliteration of the septum of an inferior second molar to receive the root of a third molar, the roots of which were coalesced. Therefore the credit for reforming the alveolus should be given to Dr. Morrison.

It may be of interest to state that previous to 1876, Dr. Morrison had replanted and transplanted over forty teeth.

LOUIS JACK.

